# Hemocyte Changes During Immune Melanization in *Bombyx Mori* Infected with *Escherichia coli*

**DOI:** 10.3390/insects10090301

**Published:** 2019-09-16

**Authors:** Tian Li, Dengfeng Yan, Xiaohui Wang, Liang Zhang, Ping Chen

**Affiliations:** 1 College of Biotechnology, Southwest University, Chongqing 400715, China; litianforever@163.com (T.L.); 18135348884@189.cn (D.Y.); zhangliang111@wo.cn (L.Z.); 2 State Key Laboratory of Silkworm Genome Biology, Southwest University, Chongqing 400715, China; 3 Key Laboratory of Sericultural Biology and Genetic Breeding, Ministry of Agriculture and Rural Affairs, Chongqing 400715, China

**Keywords:** hemocytes, melanization, immunity, silkworm

## Abstract

Hemolymph melanization is a conserved immune response in insects and other arthropods. However, the physiological process of the hemolymph system in the melanization response is hardly studied. Here, alterations of hemocytes in immune melanization were observed by *Escherichia coli* infection in *Bombyx mori*. Results first showed that there were cells aggregating into clusters. However, it vanished, and only part of clustered hemocytes were melanized during the period of intense immunity. The hemocyte numbers immediately decreased following an immune challenge, slowly increased to a peak, then reduced and finally returned to normalization. Granulocytes participated in cells aggregation at the early and later immune stage, while plasmatocytes were responsible for hemocytes agglomerate and melanization for the longest time, and more oenocytoids appeared at the peak stage of melanization. Moreover, hemocytes played a crucial role in resisting invasion of pathogens by agglomerate and melanization, and the circulatory system maintained higher hemocyte numbers and stronger antibacterial activity in fifth than fourth instar larvae after infection. In vitro immune melanization was most likely preferentially implemented in an independent process. These were the main characteristics reflecting the physiological process of hemolymph immune melanization, which provided an important foundation for further study of the complete mechanisms in the immunity of silkworm.

## 1. Introduction

**Figure 1:**
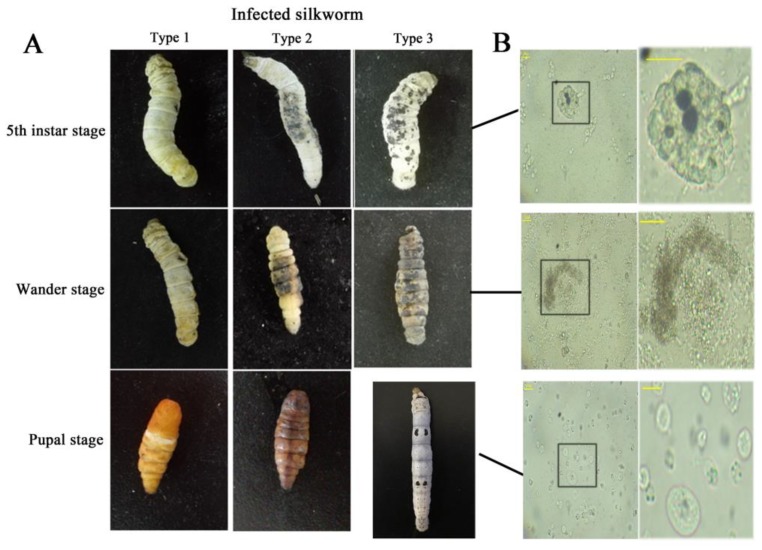
(**A**) The development of disease silkworm with melanin deposition on integument. Type 1: larvae with less pigment on the integument metamorphosed normally to pupae with the normal phenotype; type 2: larvae with more pigment pupated with a black body color, and died soon after pupation; type 3: larvae with the most pigment turned black, limp and died before pupation. (**B**) Hemocytes morphology corresponding to the larvae states in (**A**). The right column provides a close-up of the areas within the black boxes corresponding in the left column. The black boxes show the areas to zoom in on. The horizontal bar “一” represents 20 μm.

Melanin is a very important kind of animal pigment [1,2]. Redundant melanin product can be caused by immune response from injury or infection, and the area of melanin reflects the extent of damage [3,4] and even lifespan in insects. For example, we see that fifth instar larvae with less pigment on the integument metamorphosed normally to pupae with the normal phenotype, while those with more pigment pupated with a black body color died soon after pupation. Larvae with the most pigment became black, limp and finally died before pupation in silkworm culture (Figure 1A). These diseased larvae hemolymph is often accompanied by melanization. Hemolymph melanization is a conserved immune response in insects and other arthropods [5,6], and is involved in many molecular and/or biochemical reactions, such as serine protease cascade, prophenoloxidase cascade, lozenge, lipopolysaccharide-binding protein and reeler, etc. [7,8,9,10,11,12,13,14,15]. However, the knowledge of the physiological process of hemolymph circulatory system, which contains various complex reactions to immune melanization, is lacking.

Insect hemocytes—as the major components of innate immune system—are not only responsible for cellular immunity, but participate in the humoral defenses by a typical factor such as antimicrobial peptides (AMPs), lectin and lysozyme, etc. as well [16,17]. Both encapsulation and nodules formation as the primary means of cellular immunity are accompanied by melanization [18,19,20,21]. In Lepidoptera, hemocytes are divided into five subsets: prohemocytes, plasmatocytes, oenocytoids, granulocytes, and spherulocytes [22]. It has been proved that oenocytoids produce the precursor of phenoloxidase (pro-PO)—which is a key enzyme for melanin—and contribute to immune melanization [23,24]. However, granulocytes are the main melanized hemocytes that engulf fluorescent beads in the *Manduca sexta (M. sexta)* larvae [25]. Silkworm sessile hemocytes form melanized nodules on the areas of hemocoel ventral to the gut and fat body [7], and granulocytes participate into the encapsulation response after the injection bead [26]. In *Helicoverpa armigera*, granulocytes, as well as oenocytoids, are capable of encapsulation and melanization by binding to a novel C-type lectin HaCTL7 through its C-terminal carbohydrate-recognition domains CRD2 [27]. This implies that the types and ways of lepidopteran hemocytes involved in immune melanization are different and need to be explored further. 

The *Bombyx mori (B. mori)* is the most important economic insect to the silk industry and is a good model of Lepidoptera as well [28]. Although some results have been published recently using genome information and modern technology, the knowledge is too scattered to systematically understand the mechanism of melanization immunity. In this paper, we observe the circulating hemocyte changes on morphology, number and type in silkworm larvae injected with *Escherichia coli (E. coli)* to display the process of hemolymph immune melanization arising, strengthening, declining and disappearing. These findings will provide a macroscopic view to understand the complete mechanisms in melanization immunity, and create a foundation for studying the order of activated hemocytes or molecules in the whole process of hemolymph immune melanization in silkworm.

## 2. Materials and Methods

### 2.1. Experimental Silkworm Rearing and Hemolymph Collection

The silkworm strains Dazao were used and maintained at the Southwest University in China and fed mulberry under standard conditions (24–26 °C and 70% to 85% RH with a photoperiod of 12:12 LD). Experimental individuals were chosen based on similar weight and by being newly exuviated within an hour. The hemolymph was collected from the proleg of larvae via puncture with a sterile needle on ice—which was surface-sterilized with 70% alcohol—and each larva was collected only once. 

### 2.2. Pathogen Preparation

The *E. coli* (BL21, DE3) with *Dsred* (red fluorescent protein) was cultured overnight at 37 °C in Luria-Bertani’s rich nutrient medium, and was then collected by centrifugation at 7000× *g* for 10 min at 4 °C and suspended with phosphate buffered saline (PBS, 8.74 g NaCl, 1.78 g NaH2PO4, 1000 mL H2O, pH 6.5) to a concentration of OD 600 ≈ 0.8 after washing twice. When the bacterial solution reached an optical density of OD 600 ≈ 0.6, isopropyl-β-d-thiogalactoside (IPTG, 1 mmol/L) was added to induce the expression of red fluorescent protein at 37 °C for 5 h. The bacteria was collected and suspended as described above in order to prepare the *Dsred*-expressing *E. coli* suspension. 

### 2.3. Silkworm Injection

Larvae were cold anesthetized and were then injected liquid using a homemade capillary syringe through the third spiracle into hemocoel to avoid bleeding as much as possible. 

### 2.4. Hemocyte Profiles Observation In Vivo

Ten μL of hemolymph from a larva with normal phenotype were collected at 0, 1, 2, 3, 6, 9, 12, 18, 24 h after injection to investigate cellular morphology and the melanization level under a light microscope after 5 min of immobilization on a hemocytometer. Four individuals were chosen at each time point, and each treatment was repeated three times. Cellular morphology was observed per units, and images were taken from representative fields. 

### 2.5. Melanization Level Measurement

The areas of melanized hemocytes were used to estimate melanization levels. Using the same magnification of the light microscope, three horizons were chosen at random for each hemolymph sample from fifth instar larvae infected 10 μL *E. coli*. Then the total areas of melanization were calculated by Via ImageJ software, and melanization level for per sample was an average of data from three horizons. 

### 2.6. Hemocyte Numbers Calculation

The 10 μL hemolymph collected were immediately treated with 10 μL of ice-cold anticoagulant buffer (186 mM NaCl, 41 mM citric acid, 98 mM NaOH and 17 mM EDTA with a few crystals of phenylthiourea, pH adjusted to 4.5) described previously [29]. Then, 10 μL of the mixture was placed on a hemocytometer to evaluate the hemocyte numbers. Hemocyte numbers were counted for three times from four units (upper left, upper right, lower left and lowed right) of nine units (each unit containing 16 medium squares) on the hemocytometer, and the total hemocyte numbers (cell/10 μL hemolymph) was the sum of cells in four units /80 × 400 × 10^4^ × 2. Four individuals were chosen to experiment at each time point and each treatment was repeated three times. 

### 2.7. Hemocytes Classification and Count

Acridine orange (2.5 μL, 10 μg/mL) and propidium iodide (2.5 μL, 2 μg/mL) were added to 10 μL of fresh hemolymph and mixed thoroughly on Parafilm for 2 min. Then, 10 μL of the mixture was dropped onto a glass slide and covered with a coverslip to distinguish cells type according to the means of Ling provided [30], and to count the number of hemocytes of different types. Four individuals were chosen at each time point and each treatment was repeated three times.

### 2.8. Survival Statistics

Five and 10 μL of *E. coli* were injected to fourth or fifth instar larvae to examine survival. The control experiment was done by injecting PBS under the same conditions, and each treatment had three replicates containing 30 individuals. The injected silkworms were placed on sterile mounting kits for mounting at 25 °C to tally dead individuals, which lasted 24 h. 

### 2.9. Hemolymph Melanization and Hemocyte Profiles Observation In Vitro

Sixty μL of fresh hemolymph were mixed with 20 μL of the *E. coli* suspension or PBS, which were incubated at room temperature to observe melanization or to assess the absorbance at 490 nm using microplate spectrophotometer (Bio-Rad) at 25 °C. Ten μL liquid from the mixture prepared as above were taken at 1, 3, 6, 9, 12 h after incubation, to observe cellular morphology after 5 min immobilization on glass slide. The hemolymph collected at least five larvae as an experiment for observation, and each treatment was repeated three times. 

### 2.10. Hemocytes Immune Process Observation In Vitro

The mixture including fresh hemolymph and Dsred-expressing *E. coli* as 6:1 (volumetric ratio) were incubated at room temperature. The cellular morphology was continuously observed and photographs were taken at 0, 1, 3, 6, 9 h after 5 min immobilization using the Olympus FV1000 confocal microscope. The collection method of hemolymph was the as same as the above observational experiment in vitro, and the experiments were repeated three times.

### 2.11. Antibacterial Activity Detection

Collected fresh hemolymph was centrifuged to separate the hemocytes from the plasma at 4000× *g* for 10 min at 4 °C. Isolated hemocytes from precipitation were suspended with PBS, in which the volume is half of the original hemolymph. The fresh hemolymph, isolated plasma, hemocytes suspension, hemolymph after 12 h in air and LB medium were mixed with *E. coli* as 3:1 (volumetric ratio), respectively. After incubation at room temperature for 0, 1, 3, 6, 9, 12 h, the mixtures were diluted 106 times and were spread on LB agar plates containing ampicillin. Then, the colony forming units in the plate were counted after overnight incubation at 37 °C. The hemolymph collected at least five larvae as an experiment for observation, and each treatment was conducted three times.

## 3. Results

### 3.1. Cytological Morphology in Larval Hemocoel After Infection

**Figure 2:**
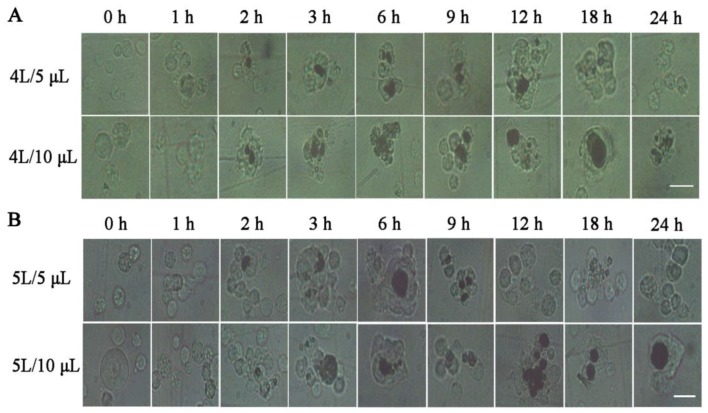
The hemocytes morphology profile in hemocoel after injection of *E. coli.* The represent times were 0, 1, 2, 3, 6, 9, 12, 18 and 24 h after injection bacteria. (**A**) 4 L/ 5 μL and 4 L/ 10 μL refer to fourth instar larvae injecting 5 and 10 μL *E. coli*, respectively. (**B**) 5 L/ 5 μL and 5 L/ 10 μL refer to the fifth instar larvae injecting 5 and 10 μL *E. coli*, respectively. The horizontal bar “一” represents 20 μm.

To observe the cytological changes in larval hemocoel after infection, the fourth and fifth instar larvae were injected with 5 or 10 μL of the *E. coli* suspension. PBS was used as the control to replace *E. coli.* We found that the first abnormality was agglomerate cells followed by a few melanized hemocytes in clustered cells. Along with the melanized hemocytes and/or agglomerate cells, which increased and then reduced gradually in sizes and numbers, the free-cells numbers changed contrarily in the hemocoel—suggesting that agglomerate cells likely originated from free-cells. In addition, melanized hemocytes decreased when *E. coli* reduced or disappeared—indicating that hemocytes melanization most likely had a function of scavenging pathogens (Figure 2). Interestingly, only some agglomerate cells were melanized, but the cell clusters with melanization vanished earlier than those without melanization—suggesting that the melanized hemocytes emerged in a period of intense immunity while agglomerate hemocytes existed in the whole course of immune response. This process—including the hemolymph circulatory system defense against *E. coli* and the return to normal—was identical in the four experimental groups. However, there were some distinctions among the experimental groups (Figure 2): the numbers of melanized hemocytes were the most at 6–9 h, but reduced significantly after 12 h in the 5 μL dose group, whereas in the 10 μL dose group melanized hemocytes were present until 24 h and the highest numbers were observed at 12–18 h in two of the instar larvae (Figure 2)—the melanization levels were listed in Supplementary Table S1. The rate at which bacteria was eliminated was faster in the fifth instar larvae compared to the fourth larvae with a 5 μL dose instead of a 10 μL dose (not shown)—suggesting that the speed of clearing pathogens correlated with both developmental stages and immunogen dosages. There were agglomerate cells, but no melanized hemocytes arising in larval hemocoel in control groups (not shown). 

### 3.2. Hemocyte Numbers in Larval Hemocoel After Infection

**Figure 3:**
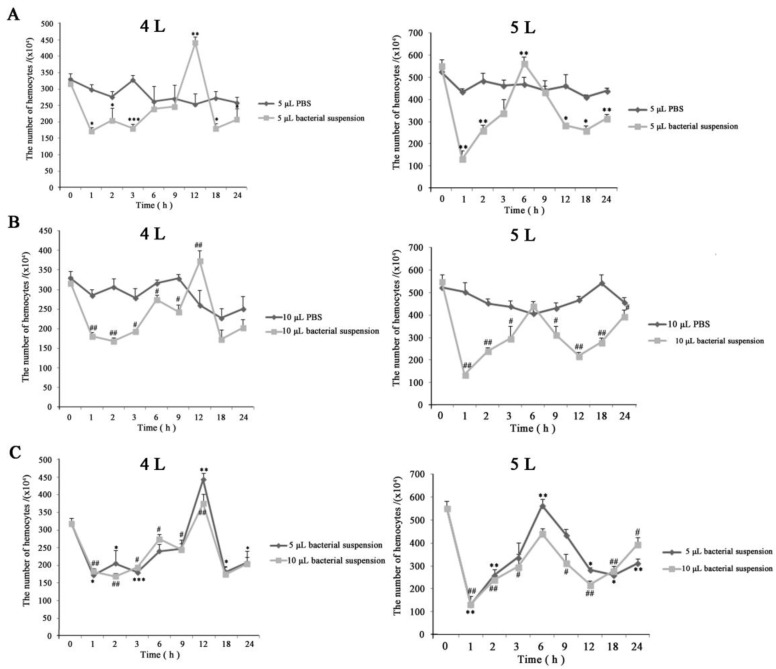
The change of hemocyte numbers in hemocoel after infection; 4 L: fourth instar larvae; 5 L: fifth instar larvae. Statistically significant differences are denoted with */# (*p* < 0.05), **/## (*p* < 0.01) or ***/### (*p* < 0.001) for injecting 5 and 10 μL, respectively. (**A**,**B**) The variations in hemocyte numbers of larvae injected PBS or *E. coli* for 5 and 10 μL, respectively. (**C**) The merged diagram of the change tendency of hemocyte numbers after injection of 5 and 10 μL *E. coli*.

**Figure 4:**
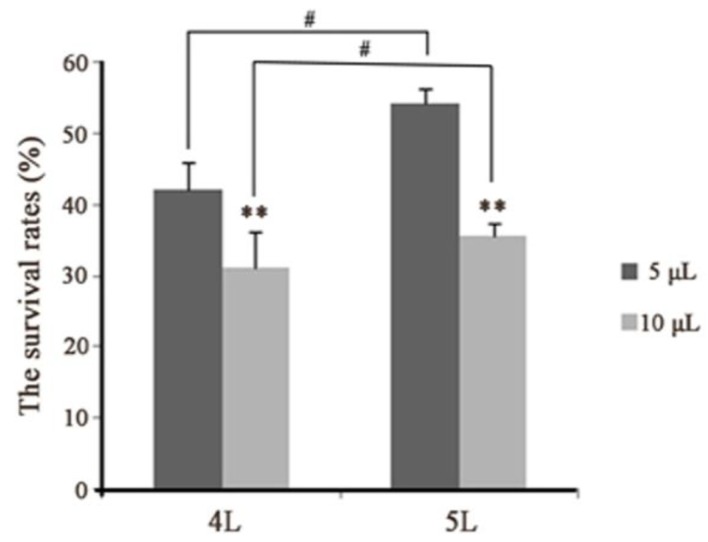
Survival of *B. mori*-injected *E. coli*.; 4 L: fourth instar larvae; 5 L: fifth instar larvae. Five and 10 μL represent the doses of *E. coli* injections. Statistically significant differences are denoted with */# (*p* < 0.05), **/## (*p* < 0.01) or ***/### (*p* < 0.001) for injection in different instar and in one instar, respectively.

The density of hemocytes (hemocyte numbers) was verified while investigating cellular morphology after injecting *E. coli*. The results showed that the abundance of hemocytes decreased immediately following an immune challenge, and began increasing slowly 1 h later. Later, the number of hemocytes peaked and then reduced again. Finally, circulating hemocytes numbers moved towards normalization—indicating the immune completion of hemolymph circulatory system (Figure 3). The trends of change were similar among the four experimental groups (Figure 3). However, between the different instars, there were differences in the numbers (about 3.16 × 106 and 2.07 × 106 in the fourth instar larvae, and about 5.52 × 106 and 3.96 × 106 in the fifth instar larvae, at 0 and 24 h after injection, respectively), the time at which cell numbers reached a peak (at 12 and 6 h in the fourth and fifth instar larvae, respectively), and the range of variation (about 1.45 × 106 and 4.18 × 106 in the fourth and fifth instar larvae from 0 to 1 h after injection, respectively) (Figure 3A,B). The least number of hemocytes was observed at 18 h in the fourth instar larvae—unlike in the fifth instar larva, in which the same phenomena was observed at 1 h. These suggested that the immunological characteristics varied between the developmental stages. Besides, the survival rates were 42% and 31% in fourth instar larvae, and 53% and 36% in the fifth instar larvae in the 5 and 10 μL group, respectively (Figure 4). Together, these results showed that in the *E. coli* infected fifth instar larvae variations in the amplitude of circulating hemocytes were higher, the changes were faster, and the recovery times were shorter compared to the fourth instar larvae—indicating that the anti-infection ability was more powerful in the fifth instar larvae.

In control groups, there was no dead individual and the hemocyte numbers were higher than that of the corresponding experimental group (Figure 3A,B). Of these, the variation ranges in cell counts were slightly larger as well in fourth instar larvae with 10 μL PBS than other controls (Figure 3B); this could be because the fourth instar larvae—with a lesser ability to maintain homeostasis—were injected relatively large doses of PBS.

### 3.3. Hemocyte Types in Larval Hemocoel After Infection

**Figure 5:**
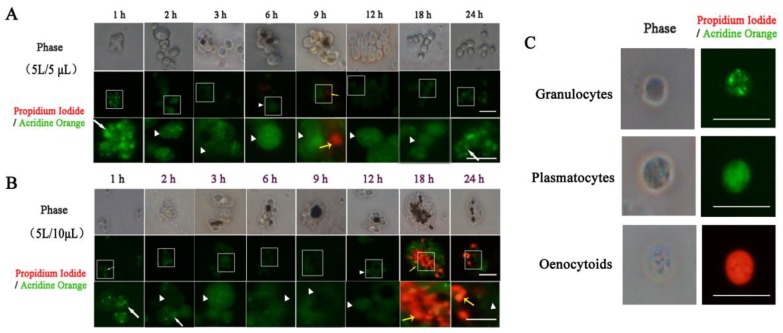
Morphology types of hemocytes using fluorescence microscopy. (**A**) 5 L/ 5 μL and (**B**) 5 L/ 10 μL refer to the fifth instar larvae injection of 5 and 10 μL *E. coli*, respectively. One, 2, 3, 6, 9, 12, 18 and 24 h represent the times after injection bacteria. The first line is the hemocyte morphology under white light, the second line is the hemocyte morphology under fluorescent light, and the third line is a local area magnification of hemocyte morphology corresponding with the second line in (**A**) and (**B**). The white boxes show the areas to zoom in on. The white arrow shows granulocytes containing bright green fluorescent granules. The white arrowhead shows plasmatocytes containing homogeneously faint green in the cytoplasm. The yellow arrowhead shows oenocytoids emitting red fluorescence (Ling et al., 2003). (**C**) Morphology of granulocytes, plasmatocytes and oenocytoids. The horizontal bar “一” represents 20 μm.

To research types of hemocytes participating in immune response, the fifth instar larvae were injected with 5 or 10 μL *E. coli*. Each treatment was repeated three times. The observation showed that during early infection stage, small cell clusters consisted of only granulocytes. With an increase in the number and size of clusters, there was a corresponding increase in the number ratio of aggregating cells of plasmatocytes. This trend lasted until hemocytes melanization was arisen with all aggregating hemocytes staining faint green except for the blackened area. Then, hemocytes melanization continued to increase until 18 h (in the 10 μL dose group). Some melanized hemocytes glowing with red fluorescence were dead oenocytoids (Figure 5). At this point, nearly all hemocytes, including clusters and individual cells, were homogeneously stained faint green—except for melanized hemocytes, indicating that plasmatocytes were the major hemocytes at this stage in the hemocoel. Because plasmatocytes as well as oenocytoids could be melanized with L-DOPA incubation in vitro [31], the unstained melanized hemocytes should be plasmatocytes (dead plasmatocytes) as well. When the melanization of the hemocytes gradually decreased and faded out completely, many agglomerated cells were still homogeneously faint green. These demonstrated plasmatocytes were the primary cells for both aggregation and melanization after melanized hemocytes appeared. At a later stage of the immune response—when the number of clusters decreased—aggregating hemocytes stained bright green, suggesting that granulocytes increased gradually to recover the hemolymph to a normal state (the percentage of major hemocyte types is listed in the Supplementary Figure S1). Because the injected dose was larger and required a longer time to dump pathogens, there were many melanized hemocytes among the aggregating hemocytes that homogeneously stained faint green and a faint red fluorescence until 24 h in the 10 μL dose group (Figure 5).

### 3.4. Hemolymph Melanization and Immunity In Vitro

**Figure 6:**
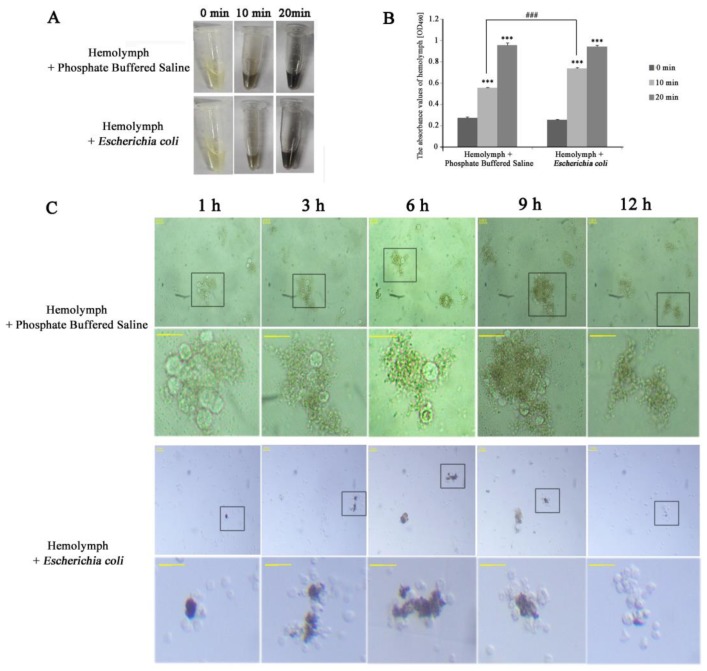
The process of melanization in vitro. (**A**) The profiles of hemolymph colors. (**B**) The profiles of hemolymph colors using a microplate spectrophotometer. Zero, 10 and 20 min represent the incubation times in the air. Statistically significant differences are denoted with */# (*p* < 0.05), **/## (*p* < 0.01) or ***/### (*p* < 0.001) compared to 0 min for each group and in 10 min between two groups, respectively. (**C**) The profiles of hemocytes morphology. The black boxes show the areas to zoom in on. The bottom-line is a local area magnification of hemocyte morphology corresponding with the top line in both groups. One, 3, 6, 9 and 12 h represent the incubating times of the mixture. The horizontal bar “一” represents 20 μm.

To study the melanization and immunity of hemolymph in vitro, *E. coli* was mixed with hemolymph for infection. We found the infected hemolymph darkened slightly slower than the uninfected hemolymph within 10 min (Figure 6A) and there was significant difference between the two groups (Figure 6B). This might be related to the hemolymph immune response induced by bacteria. Microscopic observation showed that hemocytes rapidly aggregated in 10 min after the breaking, and hemocyte clusters turned brown-black (not shown). Aggregation peaked at 1 h, and at 12 h few cells were visible—indicating that duration was the likely reason for the blackening of the hemolymph. When hemolymph was mixed with *E. coli*, the cell cluster was the most at 9–12 h, started rupturing at about 12 h and completely disappeared at 24 h (Figure 6C). The speed of aggregation and collapse was obviously slower in infection hemolymph, suggesting that the immune response could slow down the coalescence and disappearance of hemocytes. This was in line with the previous experiment, where the infected group darkened slower than the uninfected group in 10 min. It is likely that immune response is a priority process in the air. Interestingly, there were melanized hemocytes only in the infection group where hemocytes melanization occurred within 1 h in aggregating hemocytes and increased along with hemocytes agglomerate (Figure 6C), which were similar to the results in vivo—indicating the hemocytes could be melanized by pathogen in vitro.

**Figure 7:**
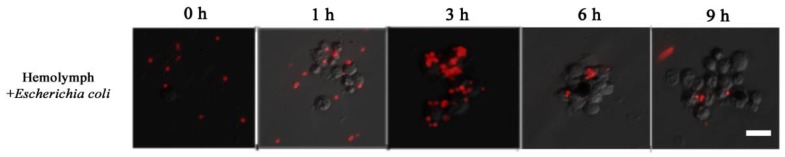
The process of immune in vitro by hemolymph mixing Dsred-expressing *E. coli.*; 0, 1, 3, 6 and 9 h represent the incubating times of the mixture. The horizontal bar “一” represents 20 μm.

Dsred-expressing *E. coli* was used to observe the process of hemolymph immune melanization at certain times in vitro. A large number of free bacteria were evenly distributed at the beginning. Later, hemocytes came closer to each other forming cluster, and wrapped around the bacteria (as 1 to 3 h in Figure 7). At about 6 h, the number of bacteria was much lower and the fluorescent signal on melanized hemocytes faded (Figure 7). These observations indicated that cell clusters play a major role in immune defense, with hemocytes melanization playing a role in dissolving bacteria. 

**Figure 8:**
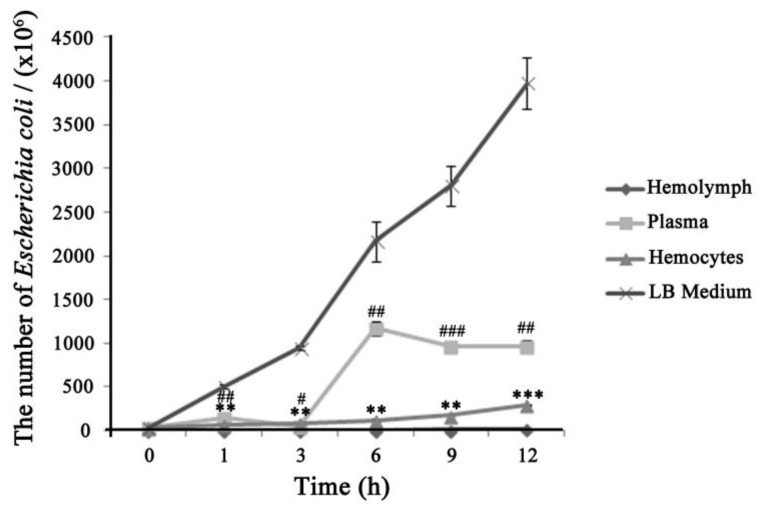
(**A**) The process of melanization in vitro. Left: the profiles of hemolymph colors; right: the profiles of hemocytes morphology. The black arrow shows melanized hemocytes. A close-up of large hemocytes melanization at 6 h is in the box of the bottom right corner. (**B**) The process of immune in vitro by hemolymph mixing Dsred-expressing E. coli. The horizontal bar “一” represents 20 μm.

To investigate hemocytes resistance to pathogens, *E. coli* was mixed with hemocytes and bacterial colonies were counted after overnight culture. Bacterial growth remained low in hemocytes, drastically inhibited in hemolymph, and multiplied rapidly within 3 h in plasma (Figure 8). These results suggested that hemocytes had stronger antimicrobial activity against *E. coli* than plasma, but had lower activity than hemolymph where hemocytes interacted with the plasma. The bacterial colony counts grew fast at later stage for the plasma group. It is possible that the killer element in plasma was initially depleted, and there was then a large amount of nutrition to promote bacteria growth. 

## 4. Discussion

Hemocytes can suppress the growth of bacteria by attracting, adhering and dissolving *E. coli* in vitro. The hemolymph displayed a weak effect on restraining bacterial growth after being exposed to air for 12 h (no hemocyte) (Supplementary Figure S2). There were no visible hemocytes in the hemolymph containing black-brown in dying larvae injected bacteria (data not shown) or in dying larvae with severe melanization (Figure 1B). These results indicate that the hemocytes play a crucial role in resisting the invasion of pathogens, and its changes are useful in reflecting the physiological process of hemolymph system in the melanization response. 

The hemocytes were uniformly distributed in the hemolymph of normal silkworms (Figure 1B). When larvae are injected *E. coli*, the cells agglomerate into clusters, and then, being melanized, emerge, enhance, or vanish—exhibiting circulating hemocytes changes on morphology in hemocoel. The clustered cells first arise as an abnormality and are then cleared; they are found in the control larvae injected with PBS as well. Some hemocyte clusters containing melanin deposition were observed in diseased silkworms (Figure 1B), and the hemolymph in vitro primary relies on cells agglomerate to kill bacteria. These data strongly illustrate hemocytes agglomeration an important defense mechanism in silkworm—which is consistent with the reports on other arthropods [21,32,33]. Besides, in vitro hemocytes melanization appears only in the infected hemolymph and the bacteria with fluorescence disappears in melanized hemocytes. Melanized hemocytes on a much larger scale appear at the prime time of immune response, and decrease when *E. coli* reduce or disappear in vivo. These reveal that hemocytes melanization have an effect on eliminating pathogens—which is consistent with previous studies in other insects [25,34]. However, only a part of clustered hemocytes that are melanized arise later, but are cleared earlier than clustered hemocytes. This is because the intermediate products (as quinines or reactive oxygen species) of the melanogenesis process could not only kill foreign pathogens—but are harmful to the hosts as well [35,36]—and as little as possible melanization is chosen for hemolymph immune defense. Therefore, hemocytes melanization can resist pathogens robustly and appears as a particular defense mechanism in silkworms.

New hemocytes are produced to replace damaged hemocytes that are needed to maintain homeostasis in organism [37]. Hemocyte numbers changed markedly in our observation, which is a result of hemolymph circulatory system removing damaged hemocytes (as the clustered cell or melanized hemocytes) and supplying new hemocytes for immune defense. The hemocyte numbers of Pieris brassicae larvae have a rapid, significant decrease after wounding (control) and oviposition for parasitized Cotesia glomerata [38]. Here, the decrease in the hemocyte numbers immediately following stimuli could likely be due to the infection wound and wound healing. Then, hemocyte counts start to increase slowly 1 h later, suggesting that hemocytes—which in normal situations may rest on the organ surface—are released into the hemocoel when they exist with pathogens. Afterwards, the number of hemocytes peak. It is likely that the demise of circulating hemocytes which kill bacteria declined, and a large number of new hemocytes supplied lead to an increase in the total cell numbers. Thus, the changes and return to normal of cell numbers is a mirror of the physiological process of hemolymph circulatory system against *E. coli*. Interestingly, the trend of changes coincided well between dose groups in the same instars, but differed for different instars, reflecting that the ability of the circulatory system to maintain homeostasis in immunity is positively associated with the developmental stage. This could be why the time required for hemocyte numbers reaching a peak was less in *E. coli*-injected fifth instar larvae in this study. Moreover, the fifth instar compared to the fourth instar larvae; the former one has higher hemocyte numbers and survival rate—as well as a faster elimination of the bacteria. These indicate that the immune capacity is parallel to hemocyte numbers. This is supported by the result of *M. sexta,* where the fifth-stage (day 0) larvae, recently having undergone ecdysis, have more numbers of circulating hemocytes, a stronger immune ability to *Photorhabdus luminescens* and a higher survival rate compared to pre-wandering fifth-stage (day 5) [39]. Therefore, increasing the circulating hemocyte is an important way by which seed-breeding for disease-resistant in economic insects (such as silkworm) is proposed. The only exception comes from the research of *Pieris brassicae*, where the later instars larvae have stronger resistance to parasitization of *Cotesia glomerata* eggs by hemocytic encapsulation, which is mainly due to high spreading ability and not the numbers of hemocytes [38].

The clustered cells and melanized hemocytes appear, increase, reduce and vanish, showing the process from infection to recovery for body’s immunity in hemocoel. The clustered cells as a defense mechanism are first activated to catch, wrap, or internalize bacteria, while the melanized hemocytes play a role on disrupting internalized bacteria. In the early or last stages of immune response, granulocytes are the major hemocytes to agglomeration, which is consistent with granulocytes, accounting for 80% of all hemocytes in normal larvae. Granulocytes are not involved in melanization during the whole immune process in our observation, which is in discrepancy with the previous report [26,40,41]. This is most likely because the factors inducing hemocytes melanization responses in insects are complex [42]. Surprisingly, plasmatocytes that comprised only about 5% of normal larvae hemocytes are confirmed as the primary source of the agglomerate and individual hemocytes when hemocyte melanization begins. There is some difference where plasmatocytes—as the most abundant hemocyte-types in many insects such as *Rhynchophorus ferrugineus*, *Galleria mellonella* and *Melipona scutellaris*—are relevant to immune melanization [40,41,43]. Plasmatocytes appear rapidly when silkworm hematopoietic organ cells are cultured [44], so mass plasmatocytes involved in hemocytes agglomeration and melanization for the longest in immunity likely come from hematopoietic organ cells in larvae. Coincidentally, the order of responsive cell types in our study is similar with that in the female of *Anopheles albimanus* where the plasmatocytes in vitro activity was followed by degranulation of granular cells [45]. Moreover, Hori [46] shows that plasmatocytes have stronger resistance to a *Nucleopolyhedrovirus* infection than other hemocytes in silkworm. Thus, plasmatocytes generated in large amounts in time are the most effective cell types against bacteria in open circulatory system for infected larvae. In addition, more oenocytoids appear at the peak stage of immunity to melanization, although oenocytoids only make up 1% of the total hemocytes in normal larvae. Since silkworm plasmatocytes in circulation could differentiate into oenocytoids [47], a sudden flood of oenocytoids for melanization likely comes from plasmatocytes—but not directly from hematopoietic organ cells in hemocoel.

In normal silkworm larvae, the hemolymph is colorless, but can be rapidly melanized in the air, which is known as spontaneous melanization coming from plasma melanization [48]. In this study, the melanized hemocytes were only found in infection hemolymph, indicating that hemocytes melanization is unique to immune defense in vitro. This is supported by Sideri’s experiment where isolated hemocytes a have a developing melanization as well when incubated with *E. coli* in Grace’s culture medium [49]. Strangely, as the speed of liquid darkened, hemocytes coalescence and disappearance were clearly slower in infected than in uninfected hemolymph in the air. Our previous study has shown that the speed of melanization in vitro is faster in infection hemolymph than in control when air is cut off effectively [50]. These results suggest that immune melanization is an independent process from spontaneous melanization and is most likely the preferred method to implement in the presence of pathogens in vitro.

## 5. Conclusions

In the paper, we characterized the main features reflecting the physiological process of hemolymph circulatory system in immune melanization, and found that plasmatocytes generated in large amounts over time are the most effective cell types against pathogens in initially. In addition, our results exhibit as well that hemocytes are crucial in resisting the invasion by agglomeration and melanization, and the immune melanization is an independent process most likely preferentially implemented in vitro, and the fifth instar larvae has higher hemocyte numbers—which is likely parallel to immune capacity. This work provides a foundation for further study of the complete mechanisms and provides new insights for understanding hemocyte types and procedures taking part in the physiological process of the hemolymph system in the melanization response in silkworm and other Lepidoptera.

## Supplementary Materials

The following are available online at https://www.mdpi.com/2075-4450/10/9/301/s1, Table S1: Hemocytes melanization levels of fifth instar larvae infected 10 μL *E. coli*. Figure S1: Major hemocyte types percentage in fifth instar larvae infected 5 (A) and 10 μL (B) *E. coli*. Figure S2: Bacteria growth by counting colony numbers after incubation.
