# Supplementary material for: Hemocyte Changes During Immune Melanization in Bombyx Mori Infected with Escherichia coli
Source: Insects. 2019 Sep 16;10(9):301. doi: 10.3390/insects10090301 (PMC6780253; doi:10.3390/insects10090301)
Supplement: Supplementary file 1 [file insects-10-00301-s001.pdf]

Supplementary Materials

Tables S1. Hemocytes melanization levels of fifth instar larvae infected 10 μL *E. coli*.

**Infection Times**	0 h	1 h	2 h	3 h	6 h	9 h	12 h	18 h	24 h
**Areas of melanized hemocytes (μm2)**	0	0	624.59 ±94.91	1309.72 ±188.80	3600.25 ±128.50	3716.64 ±69.49	4497.53 ±265.04	3985.49 ±188.93	3197.57 ±148.32

Figure S1

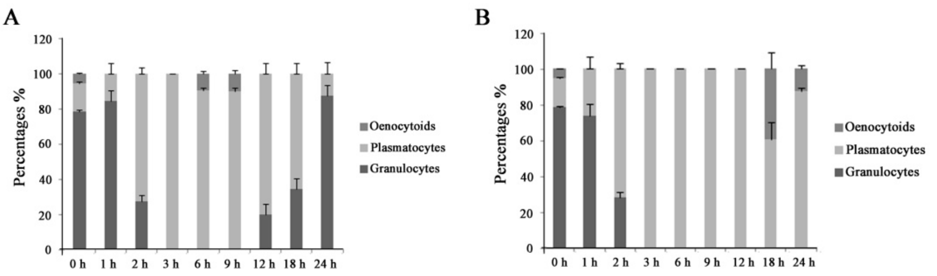

Figure S1. Major hemocyte types percentage in fifth instar larvae infected 5 μL (**A**) and 10 μL (**B**) *E. coli*. 0 h, 1 h, 2 h, 3 h, 6 h, 9 h, 12 h, 18 h and 24 h represent times after injection bacteria. Values are shown as mean + SD. (n = 4)

Figure S2

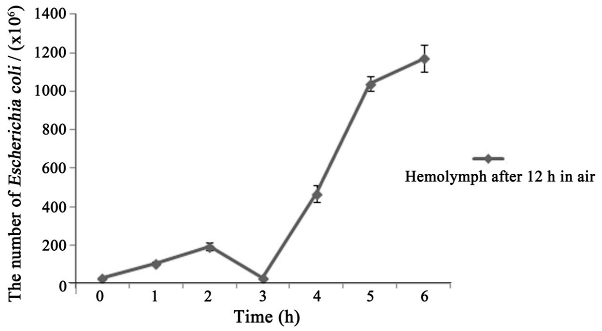

Figure S2. Bacteria growth by counting colony numbers after incubation. The colony numbers are mean ± SD for 3 independent determinations. "Hemolymph after 12 h in air" represents the mixture of E. coli and hemolymph after 12 h in air.
